# Multi-Classification of Complex Microseismic Waveforms Using Convolutional Neural Network: A Case Study in Tunnel Engineering

**DOI:** 10.3390/s21206762

**Published:** 2021-10-12

**Authors:** Hang Zhang, Jun Zeng, Chunchi Ma, Tianbin Li, Yelin Deng, Tao Song

**Affiliations:** 1 State Key Laboratory of Geohazard Prevention and Geoenvironment Protection, Chengdu University of Technology, Chengdu 610059, China; zhanghang_nn720@163.com (H.Z.); zengjun@stu.cdut.edu.cn (J.Z.); ltb2008@139.com (T.L.); Deng_YL7@163.com (Y.D.); songtao@stu.cdut.edu.cn (T.S.); 2 Chongqing City Construction Investment (Group) Co., Ltd., Chongqing 400023, China; 3 Key Laboratory of Transportation Tunnel Engineering, Ministry of Education, Southwest Jiaotong University, Chengdu 610031, China

**Keywords:** microseismic waveforms, multi-classification, convolutional neural network, similarity

## Abstract

Due to the complexity of the various waveforms of microseismic data, there are high requirements on the automatic multi-classification of such data; an accurate classification is conducive for further signal processing and stability analysis of surrounding rock masses. In this study, a microseismic multi-classification (MMC) model is proposed based on the short time Fourier transform (STFT) technology and convolutional neural network (CNN). The real and imaginary parts of the coefficients of microseismic data are inputted to the proposed model to generate three classes of targets. Compared with existing methods, the MMC has an optimal performance in multi-classification of microseismic data in terms of *Precision*, *Recall*, and *F1-score*, even when the waveform of a microseismic signal is similar to that of some special noise. Moreover, semisynthetic data constructed by clean microseismic data and noise are used to prove the low sensitivity of the MMC to noise. Microseismic data recorded under different geological conditions are also tested to prove the generality of the model, and a microseismic signal with *M_w_* ≥ 0.2 can be detected with a high accuracy. The proposed method has great potential to be extended to the study of exploration seismology and earthquakes.

## 1. Introduction

As a new real-time monitoring technology of rock mass stability, microseismic monitoring technology has been extensively applied in tunnel, mines, slopes, and other dynamic disaster early warning system projects [1,2,3,4,5,6,7,8,9]. This method can help effectively evaluate the current fracture status of surrounding rocks by analyzing the microseismic data recorded during monitoring, and then help further evaluate and predict the potential risk areas of rock masses. This is conducive to the early warning of disasters and auxiliary construction. Given the complexity of a construction environment, lengthy construction period, and continuous real-time data acquisition in tunnel projects, the various types of recorded data are often subject to interference from different background noise, including micro-fracture signal (MS) (generated by surrounding rock fractures and movement), blast, mechanical, and other unknown noise. Hence, effectively detecting the MS is challenging. MS detection depends on experience and seismic knowledge of personnel; the detection process is time-consuming and inefficient, and its accuracy cannot be ensured. Moreover, some special noise is similar to MS in time domain, which brings great challenges to MS detection. Finally, an inaccurate MS detection may make the microseismic catalog confusing and affect further analyses.

Recently, various automatic algorithms for microseismic/seismic signal detection have been proposed to resolve the above issues, such as short and long-term average (STA/LTA) [10], waveform autocorrelation, cross correlation, and fingerprint and similarity threshold (FAST) methods. Despite their advantages, each method has some disadvantages. The STA/LTA method easily misses the target signals with a low signal-to-noise ratio (SNR) [11,12]. Waveform autocorrelation, known as template matching, requires a tremendous amount of computation when the number of templates increases [13]. Although the FAST method performs well in terms of detection sensitivity and applicability, it has considerable overhead in memory and computation [14]. With the rapid development in the field of computers, artificial intelligence technology has been widely used in seismic/microseismic processing and disaster prediction [15,16,17,18]. Xin et al. (2021) [19] proposed an explainable time-frequency convolutional neural network (CNN) to provide an excellent classification performance and explainability. Liang et al. (2021) [20] combined multiple base learners and classifiers to estimate the probability of short-term rockburst risks and achieved good performance. Saad and Chen (2020) [21] extracted waveforms from continuous microseismic data using an automatic unsupervised method, which outperformed the simple k-means and short-term and long-term average ratio methods. Tang et al. (2020) [22] proposed a modified CNN with attention mechanism to detect microseismic events.

In this study, a CNN is established for the multi-classification of microseismic waveforms in frequency domain. The Short Time Fourier Transform (STFT) technology is used to transform the microseismic data in time domain to frequency domain, and a combination of the time-frequency coefficients is generated as input to the microseismic multi-classification (MMC) model. Microseismic data are divided into three types (MS, blast, and noise) as the categories of targets. The microseismic data recorded from the Grand Canyon tunnel of Lehan Expressway (China) are used for network training, validation, and testing. Compared with existing methods, the performance of the MMC is evaluated based on three metrics: *Precision*, *Recall,* and *F1-score*. Semisynthetic data are used to evaluate the noise sensitivity of the model. The proposed method is applied to test some special noise whose waveform is similar to that of MS with a low amplitude. The proposed method has been also applied in other projects under different geological conditions and engineering situations.

## 2. Method and Data Preparation

The STFT technology, also known as the windowed Fourier transform, is an effective time-frequency analysis method, whereby the time-frequency information of different time windows can be obtained by a moving window function and by performing Fourier transform in this window [23,24,25,26]. The nonstationary signal is regarded as the superposition of a series of short-time stationary signals.

(1) $$TFT\left(f,k\right)=\overset{N-1}{\underset{n=0}{\sum }}S\left(n\right)\left[W\left(n-k\right)e^{\frac{f2\pi fn}{-N}}\right]$$

where *N* and *n* represent the length of the time point of the recorded signal and time point, respectively. *S*(*n*) represents the microseismic data in time domain, and *W* is the moving window function. *K* and f represent the index of the different time windows and frequency, respectively. The length of the time window was set to 256 time points, and the window function of ‘*hann’* was selected in this study [27].

**Figure 1:**
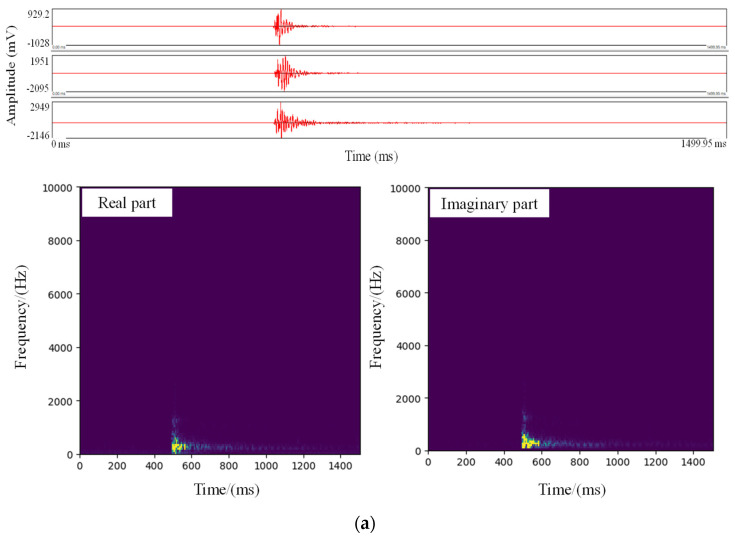

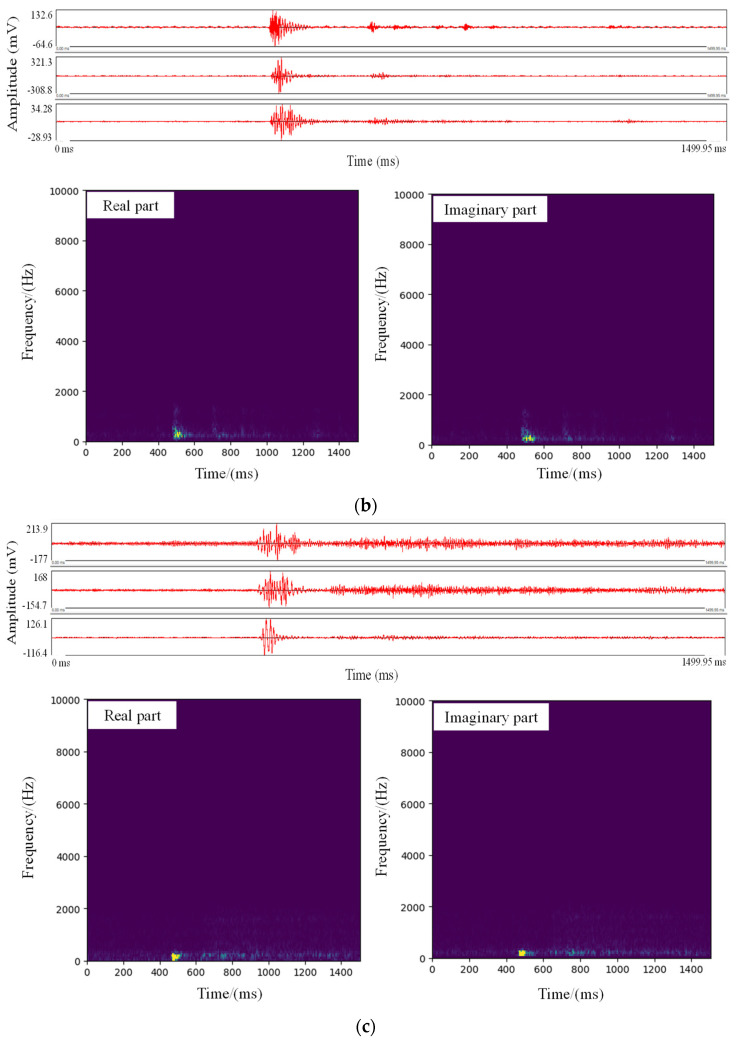
MS and similar noise data and their amplitude spectra in the Grand Canyon Tunnel. (**a**)–(**c**) MS at high and low amplitudes, and similar noise. Their amplitude spectra corresponding to the sensor are 0.1, 0.5, and 0.01 for (**a**)–(**c**), respectively. (**a**) MS with high amplitude; (**b**) MS with low amplitude; (**c**) Similar noise.

Microseismic data are typically collected and recorded by sensors (accelerometers or speedometers) in the microseismic monitoring system. Each sensor represents a channel for recording a waveform. In this work, different types of recorded data were obtained from the microseismic monitoring system installed in the Grand Canyon tunnel of Lehan Expressway (China), which is currently the deepest buried expressway tunnel in the world. The system comprises six mono-axial accelerometers with a sensitivity of 28 V/g and a response frequency ranging from 50 Hz to 5 kHz, one data acquisition station with a sampling frequency of 20 kHz, and a data processing station. The recorded data consist of 30,000 time points in voltage. Figure 1 shows the different types of microseismic data, including MS, noise, blast, mechanical, and unknown signals. Different propagation media, sensor array, and noise pollution can lead to different amplitudes of each channel in the microseismic data. In addition, some channels may not record the signal due to some technical issues.

**Figure 2:**
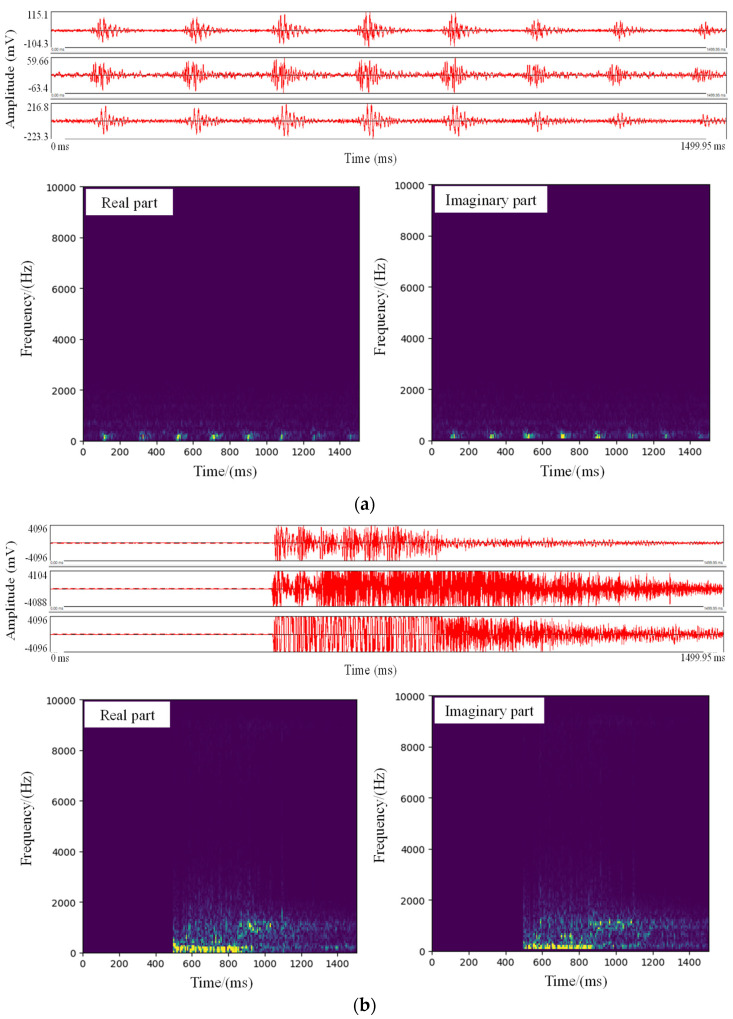

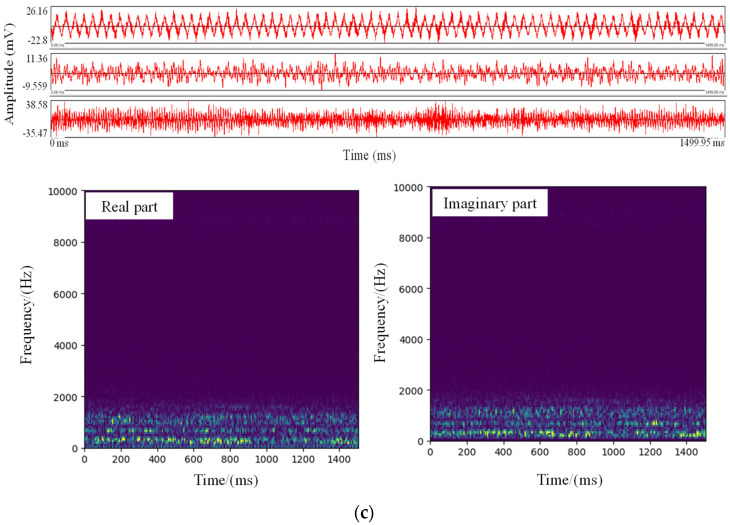
Other various types of noise data and their amplitude spectra in the Grand Canyon Tunnel. (**a**)–(**c**) Mechanical, blast, and unknown signals. Their amplitude spectra corresponding to the sensor are 0.05, 1.0, and 0.005 for (**a**)–(**c**), respectively. (**a**) Mechanical signal; (**b**) Blast signal; (**c**) Unknown signal.

Generally, microseismic data can be broadly classified into different types in time domain (Figure 1 and Figure 2). In particular, some noise waveforms (defined as similar noise) are highly similar to that of MS with a low amplitude, which brings challenges when distinguishing these two types of microseismic data in time domain (Figure 1b,c). Therefore, the time-frequency characteristics of the microseismic data are analyzed using the STFT, including the real and imaginary parts of the time-frequency coefficients (Figure 1 and Figure 2). It can be found that the frequency range and amplitude spectra have a significant difference between the different types of the microseismic data. Figure 2a shows that the blast signal covers a wide range of frequencies, and the intensity and the amplitude spectra are the highest. Its peak amplitude is mostly over 4000 mV. The intensity and frequency of the MS are relatively lower than those of the blast signal, and the waveform attenuation is faster (Figure 1a,b). The similar noise has a low frequency range and amplitude spectra, which shows an evident difference from the MS with a low amplitude (Figure 1c). Mechanical signals typically show the characteristics of regular and repeated vibrations (Figure 2b). In addition, recorded data may contain some unknown signals with unapparent features and patterns, and their amplitude spectra is the lowest (Figure 2c). Thus, different types of microseismic data can be effectively distinguished in frequency domain by the STFT.

The MS is the signal of interest for rockburst early warning, and it must be detected. The blast signal has accurate onset time picking, and the wave velocity model of the surrounding rock can be improved based on the measurable initial blast point and regression method (such as the least squares method). Combined with the improved velocity model and microseismic sensor array, it is conducive to the high accuracy of source localization. As for the other types of signals, they are useless and unnecessary. Therefore, the microseismic data can divide into three types in this study: MS, blast signal and noise. Too few samples will lead to overfitting and poor performance of the model, on account of which the various and complex characteristics of all categories cannot be covered. For the experiment in this study, 1600 MS samples, 1200 blast samples, 1500 noise samples (including 500 similar noise, 400 mechanical, and 600 unknown samples) were selected, and randomly split into two parts: training (80%) and test (20%) datasets. Each sample includes six waveforms based on the microseismic monitoring system. Moreover, the k-fold cross validation was introduced to avoid overfitting and to find the optimal model. The training dataset was divided into k parts (i.e., folds), and each fold was used as a validation dataset in turn; the remaining k-1 folds were taken as the training dataset. The model was trained k times, and the optimal model was obtained based on the training results. In this study, the k value was set to 5 to ensure that the number of microseismic waveforms of each fold was greater than 4000. The test set was mainly used to record the network performance.

## 3. Network Architecture and Training

**Figure 3:**
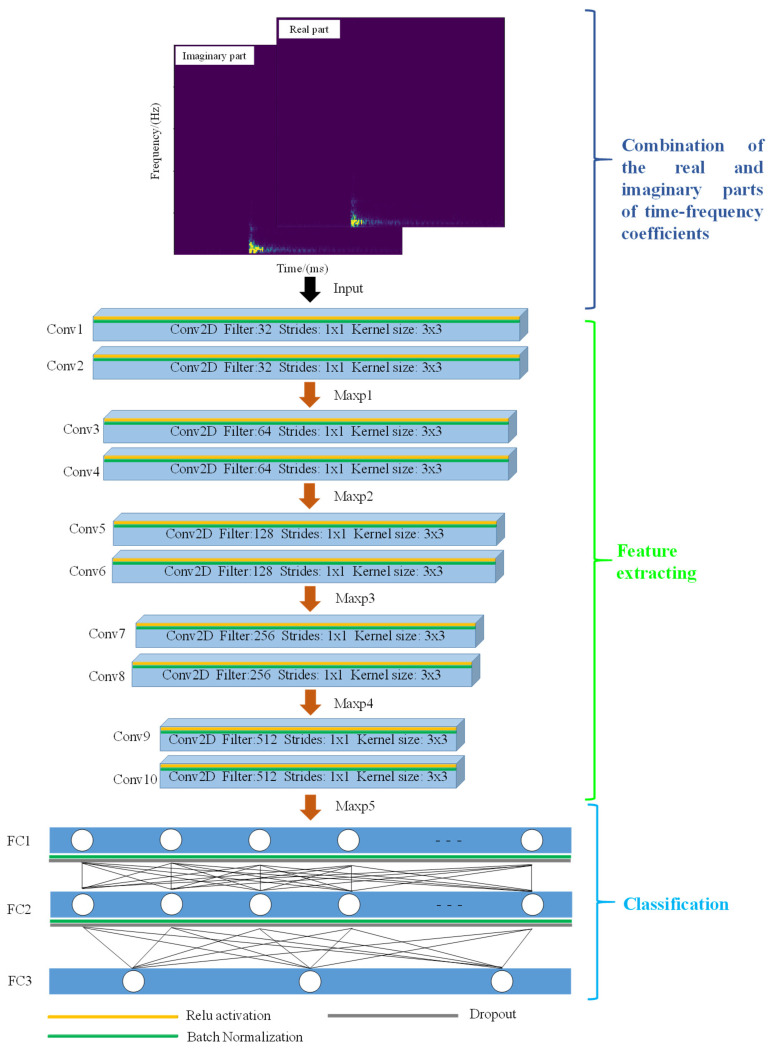
Architecture of the proposed network. It includes three parts: (1) combination of real and imaginary parts of the time-frequency coefficients as input; (2) feature extraction. The rectangular block and arrow represent the convolutional layer and maximum pooling layer, respectively; (3) classification. The output is the probability of the target category. The blue region and circles represent the fully connected layer and neurons, respectively. The other different colors of the rectangles represent different operations, including ReLU activation, batch normalization, and Dropout. Conv, Maxp, and FC represent the 2D convolution, Max pooling, and fully connected layers, respectively.

Figure 3 shows the architecture of the proposed neural network, which includes Input, convolutional layer, maximum pooling layer, flatten layer, fully connected layer, and Output. The combination of the real and imaginary parts of the time-frequency coefficients forms the network input with dimensions of 129 × 236 × 2 by applying the STFT to the signal in time domain. A series of convolution and pooling operations was used to extract and compress the input features. The kernel and stride sizes of the convolutional layer were set to 3 × 3 and 1 × 1, respectively, to extract the features of the real and imaginary parts of the time-frequency coefficients. Moreover, the maximum pooling layer with a kernel size of 2 × 2 and a stride of 1 × 1 were selected to compress the extracted feature, which helped remove the redundant information and retain the key features. Moreover, a BN operation and ReLU activation function were used to process the features after the convolution operation. The input for each layer was uniformed to accelerate the convergence and avoid the overfitting of the model based on the BN operation [28]. The ReLU activation function was proposed by Glorot et al. (2011) [29]:

(2) $$f\left(x\right)=\{\begin{array}{c} x\\ 0 \end{array}\begin{array}{c} x>0\\ x\leq 0 \end{array}$$

The outputs of zero for some neurons in Equation (2) are conducive to enhance the sparsity and nonlinear relationship of the neural network and further alleviate model overfitting. The Dropout operation is used to improve the generalization ability of the neural network and prevent overfitting by stopping the activation of some neurons with a probability [30]. The deeper the network, the greater the number of features extracted. After multiple 2D convolution and maximum pooling layers, the Flatten layer is used to convert the features into 1D vectors. Next, fully connected layers are used to perform high-level reasoning and map the learned features to the probability of the required output classes from the last step. A SoftMax activation function is used in the last layer of the network to output a vector of the predicted probabilities of each class. Moreover, the Adam optimizer for weight updates [31] and a cross entropy loss function are used. The Early Stopping operation also helps avoid the model overfitting. The learning rate is set to 0.005 he batch size to 32.

Table 1 shows the parameters of MMC model, including the layer output, activation function, kernel size, stride size, weight, and bias. Overall, the network comprises 13 layers and has 5.79 × 10^6^ trainable parameters. In this study, one-hot encoding was used for the three desired classes in the training process, and the number of epochs was set to 300.

## 4. Results

### 4.1. Model Evaluation

The structure of the MMC with 13 neural layers is similar to that of the baseline neural network VGG13, which is commonly used in image classification tasks. Therefore, the standard VGG13 and VGG16 networks were selected for a comprehensive comparative analysis of the MMC. For a fair model comparison, the parameters of the fully connected layers in VGG13 and VGG16 were set the same as those of the MMC. The same training datasets were used to train VGG13 and VGG16. The indicators of accuracy and loss are typically used to monitor the training performance of the model. A high accuracy and low loss indicate that a model has a good training effect. Table 2 shows the comprehensive comparison results of VGG13, VGG16, and MMC using k-fold cross validation. With the deepening of the neural network, the performance of the model is improved, however, the number of parameters and calculation cost (i.e., GFLOPs) are relatively increased. In addition, the complexity of the neural network affects the model performance based on the comparison between VGG13 and MMC, even if they have the same number of neural layers. Therefore, the MMC was selected for multi-classification of the complex microseismic waveforms based on the comprehensive consideration of the computing consumption, memory footprint, and model performance.

**Figure 4:**
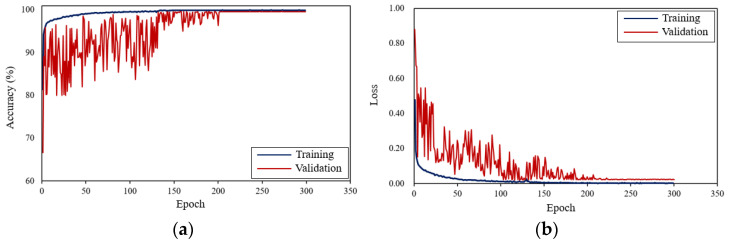
Optimal results of model training. (**a**) Accuracy changes with the epochs; (**b**) Loss changes with the epochs. The dark blue and red lines represent the accuracy and loss for the training and validation datasets, respectively. The accuracy increases with the increase in the number of epochs, while the loss decreases.

Figure 4 shows the optimal values of the accuracy and loss in the model training of the MMC using k-fold cross validation. The accuracy and loss do not change significantly in the last 90 epochs, indicating that the model gradually approaches to fitting and well trained. Finally, the accuracies of the training and validation are 99.8% and 99.5%, and the loss values are 0.009 and 0.018, respectively. These results prove that the MMC has a good performance of model training.

The test dataset (including 320 MS, 240 blast and 300 noise samples) is also used to compare the existing methods (correlation [32] and AlexNet [30]) with the MMC in terms of their performance for the multiclassification of microseismic signals. Moreover, *Precision*, *Recall*, and *F1-score* are introduced to evaluate the performance of these methods:

(3) $${\mathrm{Precision}}_{i}=\frac{{\mathrm{TP}}_{i}}{{\mathrm{TP}}_{i}{+\mathrm{FP}}_{i}}\;$$

(4) $${\mathrm{Recall}}_{i}=\frac{{\mathrm{TP}}_{i}}{{\mathrm{TP}}_{i}{+\mathrm{FN}}_{i}}\;$$

(5) $${\mathrm{Micro}\;F1-\mathrm{score}}_{i}=2\times \frac{{\mathrm{Precision}}_{i}{\times \mathrm{Recall}}_{i}}{{\mathrm{Precision}}_{i}{+\mathrm{Recall}}_{i}}\;$$

(6) $$\mathrm{Macro}\;F1-\mathrm{score}=\frac{\sum_{i=1}^{i=n}{\mathrm{Micro}\;F1-\mathrm{score}}_{i}}{n}$$

where *i* represents the category of the target. *TP*, *FP*, and *FN* are the true positives, false positives, and false negatives, respectively. *Precision* is defined as the proportion of correct predictions in the predictions that are positive (both *TP* and *FP*), and *Recall* is defined as the proportion of correct predictions in the actual positive samples (both *TP* and *FN*). *F1-score* is used to evaluate the comprehensive performance of the models and eliminate the impact of sample size imbalance [33]. *Micro F1-score* represents the performance of the method on each category, whereas *Macro F1-score* represents the comprehensive performance on all categories. Generally, the higher the *F1-score*, the better the performance of the model. For the correlation method, a large amount of waveform templates is used to provide maximum coverage for the feature information and further ensure the classification accuracy. Table 3 and Table 4 show the experimental results of the different methods. The MMC can detect 1924 MS waveforms, 1886 of which are true positive, thus outperforming the correlation and AlexNet methods. Moreover, the MMC can detect all the blast waveforms completely and accurately. The results also show that the Correlation method takes more time for the test dataset than the AlexNet and MMC. A well-trained model can efficiently deal with high-volume data and reach sufficient accuracy. In conclusion, the best performance in terms of *Precision*, *Recall* and *F1-score* indicates that the MMC can effectively extract the features of microseismic data in frequency domain.

**Figure 5:**
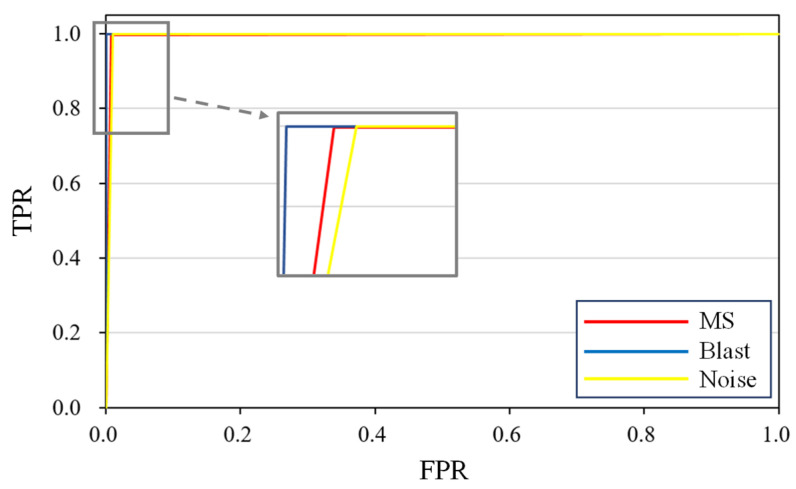
ROC curve of different classes obtained by applying the MMC to the test dataset. The AUC values of the three classes (MS, blast, and noise) are 0.995, 0.998, and 0.994, respectively.

The receiver operating characteristic (ROC) curve is introduced for the model evaluation; it represents the relationship between the true positive rate (TPR) and the false positive rate (FPR) of the classifier. The area under curve (AUC) is defined as the area enclosed by the coordinate axis under the ROC curve, and the AUC value of an ideal classifier is 1. The closer the AUC value is to 1, the better the performance of the classifier. Figure 5 shows the ROC curve of the three target classes (MS, blast, and noise). Each class is set to positive and the rest to negative. Thus, the multi-classification is transformed into binary classification, and the ROC curve and AUC value of each class can be calculated. A high AUC value of each class means that the MMC has good performance for the multiple classification of the microseismic waveforms.

**Figure 6:**
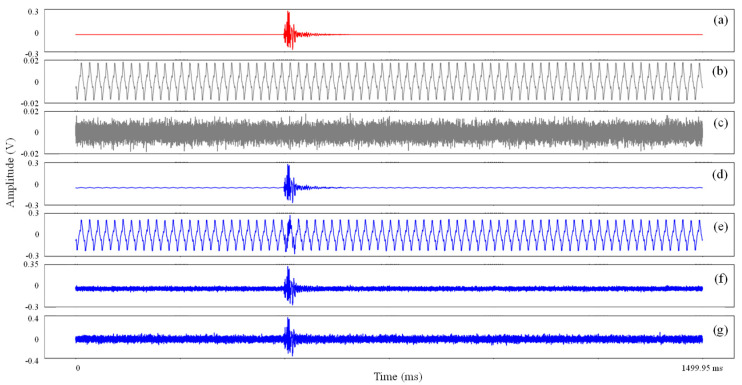
Semisynthetic data with different SNR values. (**a**) Clean microfracture waveform; (**b**,**c**) Background and Gaussian noises, respectively; (**d**,**e**) Semisynthetic data with SNR values of 35.2 and–1.9 based on background noise; (**f**,**g**) Semisynthetic data with SNR values of 10.8 and 3.9 based on Gaussian noise.

To further evaluate the noise sensitivity of the model, semisynthetic data were constructed based on clean data and noise (including background and Gaussian noises). Noisy signals with different SNR values were generated by scaling the noise amplitude (Figure 6). The detail calculation of the SNR is as follows [33]:

(7) $$\mathrm{SNR}=20\times \log_{10}\left(S_{Amax}/N_{Amax}\right)$$

where $S_{Amax}$ and $N_{Amax}$ are the peak amplitudes of the signal and noise, respectively.

**Figure 7:**
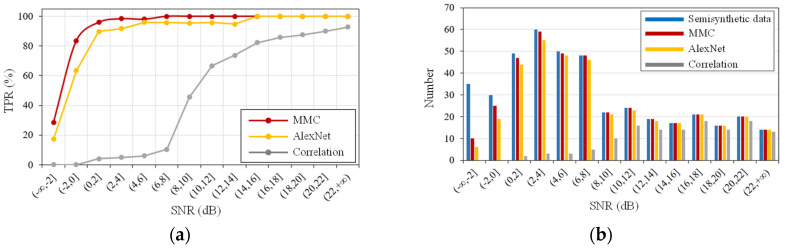
Noise sensitivity evaluation of MMC, AlexNet, and Correlation methods. (**a**) TPR (True positive rate); (**b**) detection results on semisynthetic data with different SNR values.

By adding the different levels of background and Gaussian noises to the clean signal, 14 types of noisy signals with SNRs ranging from −2 to 22 dB were formed. The MMC, AlexNet and Correlation methods were applied to these semisynthetic data. Regardless of the method used, the detection rate increases with the improvement in the SNR, and the MMC exhibits the best detection performance among these methods (Figure 7). When the SNR is close to 0, the detection rate of the model can reach more than 80%, while those of the AlexNet and Correlation are 63.3% and 0, respectively. Moreover, the MMC can completely and accurately detect the microseismic signals with a SNR higher than 2 dB. Therefore, the MMC is less sensitive to background and Gaussian noises.

### 4.2. Application and Discussion

**Figure 8:**
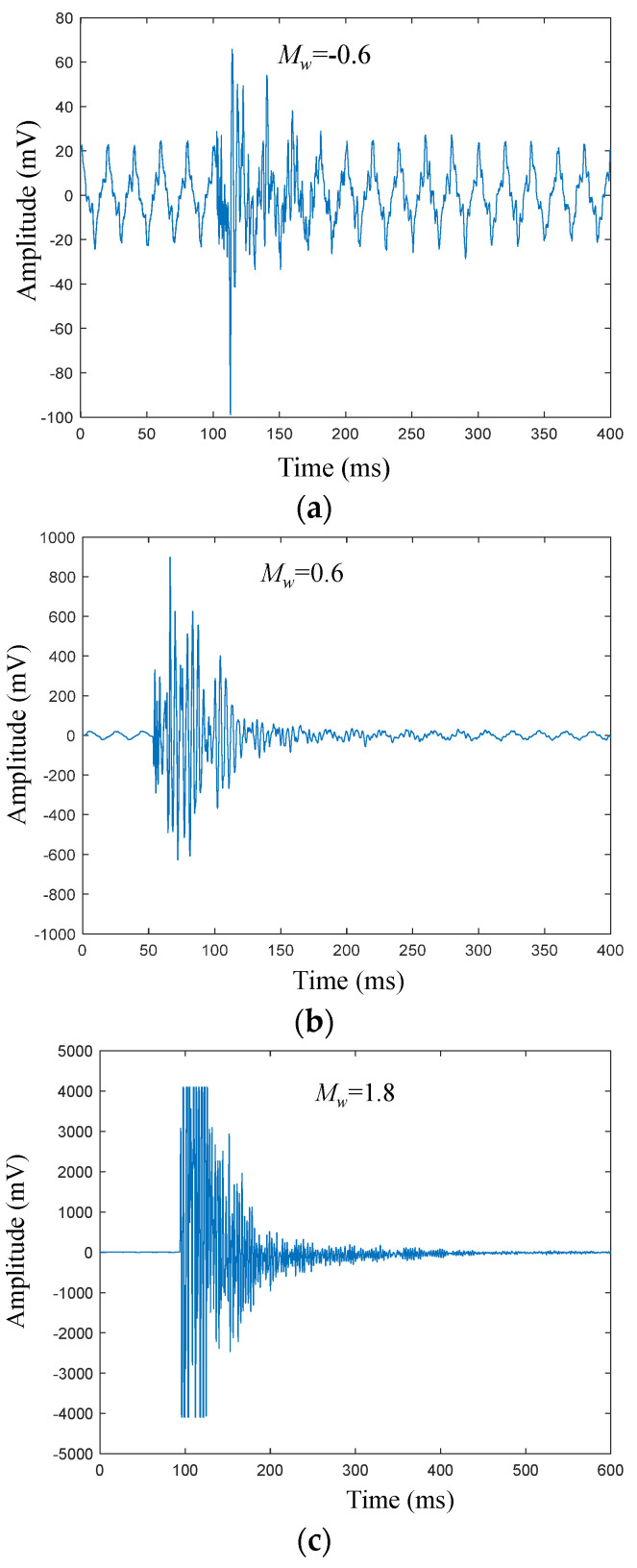
MS with different *M_w_* in Micang Mountain tunnel, China. (**a**)–(**c**) Waveforms of the channel with the highest amplitude. (**a**) *M_w_* = −0.6; (**b**) *M_w_* = 0.6; (**c**) *M_w_* = 1.8.

**Figure 9:**
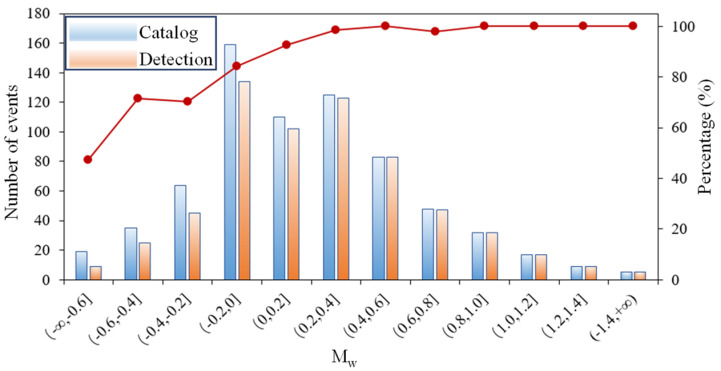
Detection results of MMC on MS with different *M_w_* in Micang Mountain tunnel.

It has been proved that the MMC can effectively classify the various types of complex microseismic data based on the training, validation, and testing. Generally, a successful model should have a good generality to deal with different situations. Hence, microseismic data recorded under different geological conditions (Micang Mountain tunnel) were applied to the proposed method. The results show that 699 MSs and 756 noise signals could be detected, of which 20 MSs and 689 noise are already found in the previous human detection catalog. The visual detection results show that 35 of the remaining MSs and 41 of the remaining noise are new, resulting in a *Precision* value of 0.937 for MS and 0.966 for noise. In addition, the moment magnitude (*M_w_*) of the detected MSs ranges from −0.6 to 1.4 (Figure 8) [34]. The MSs with *M_w_* ≥ 0.2 can be better detected by the MMC, however, it could be a challenge to detect MSs with low *M_w_* (Figure 9). For the blast signal, all the samples were correctly detected, which confirms the high performance of the proposed method in blast signal detection.

From Figure 1, we find that the MS with a low amplitude is similar to similar noise (defined in Section 2). To further evaluate the performance of the MMC on this issue, 50 MSs with low amplitudes and 50 similar noise samples were used. *Precision*, *Recall* and *F1-score* were also selected to measure and compare the performance of the different methods. The results show that the MMC outperforms the Correlation and AlexNet methods, indicating that it is more suitable for classifying complex microseismic waveforms (Table 5). Therefore, the MMC has good application prospects for the multi-classification of microseismic data in tunnels, even when some special noise is similar to MS.

The MMC can well classify microseismic data into three types (MS, blast, and noise) in frequency domain, even when the waveform of the MS with a low amplitude is similar to that of some noise. Although the proposed method has a good performance for MS detection in actual field, it has some limitations. Microfracture events with low Mw and MS heavy polluted by noise are not conducive to the accurate detection by the proposed method. Insufficient number of samples or some specific samples cannot cover the general characteristics of the target category, which can easily cause model overfitting and ineffective training. The complex monitoring environments and waveform propagation bring various types of waveforms, including natural earthquakes, rock mass ruptures, collapses, blast, mechanical, and artificial noise. It is insufficient to divide the microseismic data into three types in some cases. In addition, many models have a ‘performance bottleneck’ in actual application, which is reflected in the difficultly of improving some metrics such as *Precision*, *Recall*, and *F1-score*. Whether this issue is due to the uneven distribution of training data or the drawbacks of the model itself remains unclear. Future research topics include adding more types of microseismic data recorded under different geological conditions and regions, and the depth and complexity of the neural network, in a bid to obtain a trained model with high generality and accuracy. Moreover, an effective multi-classification of microseismic data can improve the further analysis of focal mechanism, source location, and disaster warning, etc.

## 5. Conclusions

This study developed an advanced signal processing method based on the CNN for the multi-classification of microseismic data. Considering the similarity in time domain between the MSs with low amplitudes and some special noise, the STFT technology was used to enhance the characteristics of various microseismic data to facilitate the classification in frequency domain. Compared with the Correlation and AlexNet methods, the MMC exhibited a better performance in microseismic multi-classification through model training, validation, and testing. The model was proven to exhibit a low sensitivity to noise based on semisynthetic data. Moreover, the MMC was applied to microseismic data recorded in different tunnels, suggesting that the model has generalization ability and good performance for MS detection in different geological backgrounds. The proposed method basically overcomes the difficulty in distinguishing between low-amplitude MS and similar noise. While this study is motivated by the need for efficient and automated microseismic signal processing, notably, the proposed method can be seamlessly extended to signal analysis for disaster estimation in geophysical and geotechnical fields, such as hydraulic fracturing, mining industry, shale-gas exploitation, and earthquakes.

## Figures and Tables

**Table 1:** Parameters of the MMC model.

Layer	Output	Activation Function	Kernel/Stride	Parameters
Conv1	129 × 236 × 32	ReLU	3 × 3/1 × 1	608
Conv2	129 × 236 × 32	ReLU	3 × 3/1 × 1	9248
Maxp1	64 × 118 × 32		2 × 2/1 × 1	0
Conv3	64 × 118 × 64	ReLU	3 × 3/1 × 1	18,496
Conv4	64 × 118 × 64	ReLU	3 × 3/1 × 1	36,928
Maxp2	32 × 59 × 64		2 × 2/1 × 1	0
Conv5	32 × 59 × 128	ReLU	3 × 3/1 × 1	73,856
Conv6	32 × 59 × 128	ReLU	3 × 3/1 × 1	147,584
Maxp3	16 × 29 × 128		2 × 2/1 × 1	0
Conv7	16 × 29 × 256	ReLU	3 × 3/1 × 1	295,168
Conv8	16 × 29 × 256	ReLU	3 × 3/1 × 1	590,080
Maxp4	8 × 17 × 256		2 × 2/1 × 1	0
Conv9	8 × 17 × 512	ReLU	3 × 3/1 × 1	1,180,160
Conv10	8 × 17 × 512	ReLU	3 × 3/1 × 1	2,359,808
Maxp5	4 × 8 × 512		2 × 2/1 × 1	0
Flatten				0
FC1	256	ReLU		1,048,832
FC2	128	ReLU		32,896
FC3	3	Softmax		387

**Table 2:** Comprehensive comparison between different networks using k-fold cross validation. The GFLOPs represents the computing power of the networks. Val_loss and Val_accuracy represent the loss and accuracy for the validation dataset, respectively.

Model	Parameters (×10^6^)	GFLOPs	Val_Loss	Val_Accuracy (%)
VGG13	10.52	0.025	0.018 ± 0.004	99.2 ± 0.5
VGG16	15.79	0.032	0.009 ± 0.005	99.5 ± 0.3
MMC	5.79	0.017	0.023 ± 0.005	99.0 ± 0.5

**Table 3:** Confusion matrix of the test dataset of different methods. The overall accuracies of the Correlation, AlecNet, and MCC methods are 78.3%, 98.58%, and 99.54%, respectively.

Correlation		**Predict**	**MS**	**Blast**	**Noise**	**Overall Accuracy**
	**Class**					
	MS		1377	102	396	78.33%
	Blast		59	1289	28	
	Noise		484	49	1376	
AlexNet		**Predict**	**MS**	**Blast**	**Noise**	**Overall Accuracy**
	**Class**					
	MS		1876	4	20	98.58%
	Blast		11	1434	3	
	Noise		33	2	1777	
MCC		**Predict**	**MS**	**Blast**	**Noise**	**Overall Accuracy**
	**Class**					
	MS		1909	0	9	99.54%
	Blast		1	1440	4	
	Noise		10	0	1787	

**Table 4:** Comparison between correlation, AlexNet and MMC methods on the test dataset containing MS, blast, and noise data, excluding the overhead runtimes (model training for MMC and AlexNet took 42 and 53 min, respectively).

**Correlation**								
**Classes**	***Precision***	***Recall***	***Micro F1-Score***	***Marco F1-Score***	***TP***	***FP***	***FN***	**Reported Runtime**
MS	0.734	0.717	0.726	0.794	1377	498	543	1 h
Blast	0.937	0.895	0.915		1289	87	151	
Noise	0.721	0.764	0.742		1376	533	424	
**AlexNet**								
**Classes**	***Precision***	***Recall***	***Micro F1-Score***	***Marco F1-Score***	***TP***	***FP***	***FN***	**Reported Runtime**
MS	0.987	0.977	0.982	0.986	1876	24	44	16 s
Blast	0.990	0.996	0.993		1434	14	6	
Noise	0.981	0.987	0.984		1777	35	23	
**MMC**								
**Classes**	***Precision***	***Recall***	***Micro F1-Score***	***Marco F1-Score***	***TP***	***FP***	***FN***	**Reported Runtime**
MS	0.995	0.994	0.995	0.996	1909	9	11	12 s
Blast	0.997	1.000	0.998		1440	5	0	
Noise	0.994	0.993	0.994		1787	10	13	

**Table 5:** Comparison between the Correlation, AlexNet and MMC methods on the test dataset containing MS and similar noise.

**Correlation**							
	***Precision***	***Recall***	***Micro F1-Score***	***Marco F1-Score***	***TP***	***FP***	***FN***
MS	0.668	0.683	0.675	0.672	205	102	95
Similar noise	0.676	0.660	0.668		198	95	102
**AlexNet**							
	***Precision***	***Recall***	***Micro F1-Score***	***Marco F1-Score***	***TP***	***FP***	***FN***
MS	0.904	0.937	0.920	0.918	281	30	19
Similar noise	0.934	0.900	0.917		270	19	30
**MMC**							
	***Precision***	***Recall***	***Micro F1-Score***	***Marco F1-Score***	***TP***	***FP***	***FN***
MS	0.945	0.970	0.957	0.957	291	17	9
Similar noise	0.969	0.943	0.956		283	9	17

## Data Availability

The raw/processed data required to reproduce these findings cannot be shared at this time as the data also forms part of an ongoing study.
